# Is it Possible to Predict Massive Bleeding in Nulliparous Women with Placenta Previa?

**DOI:** 10.1055/s-0040-1721355

**Published:** 2021-01-29

**Authors:** Gokcen Orgul, Sule Goncu Ayhan, Gulhan Cetinkaya Saracoglu, Aykan Yucel

**Affiliations:** 1 Department of Perinatology, University of Health Sciences, Ankara, Turkey

**Keywords:** cesarean section, nulliparity, placenta previa, postpartum hemorrhage, uterine bleeding

## Abstract

**Objective** We evaluated risk factors to determine if there were specific risk factors that could predict massive bleeding in nulliparous women with placenta previa.

**Methods** The participants were classified into two groups. Women with a calculated blood loss ≥ 1,000 mL were included in the massive bleeding group. Women without any signs or symptoms related with hypovolemia or with a calculated bleeding volume < 1,000 mL were categorized into the non-massive bleeding group.

**Results** There were 28 patients (40.6%) with massive bleeding and 41 cases (59.4%) with non-massive bleeding. The calculated blood loss and number of cases that required red cell transfusions were statistically different between the groups (< 0.005 and 0.002, respectively). There were no statistically significant differences in terms of maternal or fetal factors, placental location, or delivery characteristics between the two groups.

**Conclusion** We could not determine the predictive features for massive hemorrhage based on clinical features, delivery features, or placental location.

## Introduction

Placenta previa refers to an abnormally placed placenta, which covers the internal ostium of the cervix. The underlying etiologic reason has not been fully understood yet. Impaired endometrial receptivity in the upper part of the uterus (previous placenta previa and caesarean section scar) and big placental volume (multiple pregnancies) are found to be the main risk factors. 1 On the other hand, some risk factors for placenta previa have also been described in the literature, such as advanced maternal age, multiparity, spontaneous or induced miscarriage, cigarette smoking, and assisted reproductive technology (ART) pregnancies. 2 3

The prevalence of placenta previa is ∼ 3 to 5 per 1,000 deliveries. 4 This obstetric issue is related with serious maternal complications and adverse fetal/neonatal outcomes. Maternal morbidity and mortality is increased in women with placenta previa, most frequently due to massive bleeding and surgical complications. 5 6 Uterotonic medications, intrauterine balloon tamponade, serious surgical interventions (uterine compression sutures, uterine/hypogastric artery ligation, etc.) and hysterectomy may be required to control the massive hemorrhage. 7

The abnormal implantation of the placenta in a nulliparous woman is interesting, and the etiology remains unclear. Moreover, these women are also at increased risk for massive bleeding during surgery. Therefore, we aimed to investigate the risk factors to predict massive bleeding in nulliparous women with placenta previa.

## Methods

We have retrospectively evaluated the data of pregnant women who delivered between September 2010 and September 2016 at the Etlik Zubeyde Hanim Women's Health Care, Training and Research Hospital, University of Health Sciences, Ankara, Turkey. The study group consisted of 69 nulliparous women with prenatally diagnosed placenta previa. The exclusion criteria were: previous delivery history (> 500 gr or > 20 ^th^ gestational week), previous uterine surgery, multiple pregnancy, and fetuses with any chromosomal abnormality or structural malformation.

Prenatal complete placenta previa diagnosis was based on the transvaginal ultrasonographic findings of the classical image of the placental tissue covering the internal cervical os totally. For cases with a placental distance to the cervical os < 20 mm, the diagnosis was as low-lying placenta previa.

Data of the maternal characteristics (age, body mass index, previous miscarriage history, and coexisting diseases), obstetric outcome (preterm delivery, intrauterine growth restriction [IUGR], fetal demise, and premature rupture of membranes [PPROM]), and neonatal outcome (birthweight, APGAR scores at 1 ^th^ and 5 ^th^ minute, and gestational week at birth) were analyzed. All cases were delivered by caesarean section. Operative interventions such as uterine compression sutures, intrauterine balloon tamponade, and uterine/hypogastric artery ligation were recorded from the files of the patients. Need for red blood cell transfusion was also assessed.

The cases were classified into two groups in terms of bleeding complications. Postpartum massive hemorrhage diagnosis was based on the criteria defined by American College of Obstetricians and Gynecologists. 8 Estimated blood loss was calculated with the following formula: calculated pregnancy volume (0.75 [maternal height X 50]+ [maternal weight X 25]) x blood volume loss percentage (predelivery hematocrit – postdelivery hematocrit/ predelivery hematocrit).Women with a calculated blood loss ≥ 1,000 mL were included in the massive bleeding group. Women without any signs or symptoms related with hypovolemia or calculated bleeding volume < 1,000 mL were categorized into the non-massive bleeding group. Estimated blood loss was calculated by using the formulas previously described in the literature. Maternal height together with weight and hematocrit levels (both predelivery and postdelivery) were used for this purpose. 9 The transfusion of each unit of packed red blood cells was accepted to raise postoperative hemoglobin and hematocrit levels by 1% (g/dL) and 3%, respectively. Estimated blood loss was calculated carefully for women who were transfused with red blood cell packages. The groups were compared in terms of the maternal and fetal characteristics described above.

The study protocol was approved by the Local Committee of Ethics of our hospital with the number of 219 (21.12.2016). The authors have complied with the World Medical Association Declaration of Helsinki regarding the ethical conduct of research. Statistical analyses were performed by using the IBM SPSS Statistics for Windows, Version 23.0. (IBM Corp., Armonk, NY, USA). The mean and standard deviation values along with the median, minimum, and maximum values were calculated, and the percentages were calculated where necessary. Normality of the data distribution was assessed with the Kolmogorov-Smirnov test. Parametric data were examined with independent two-sample or *t* -test, and non-parametric data were compared using Mann Whitney-U test. Percentages were compared with chi-squared test. A *p* -value < 0.05 indicates statistically significant differences.

## Results

The total number of deliveries were 93,045 during the study period. Placenta previa was diagnosed in 395 women, with a rate of 0.42%. Among all placenta previa cases, there were 69 nulliparous women (17.4%), who were included in the study group. There was no definitive diagnosis of placenta accreta spectrum disorders in these cases.

Among all cases, 73.9% ( *n* = 51) were primigravida, while the remaining patients had at least one miscarriage history. The number of previous miscarriages was once in 12 cases (17.4%), twice in 3 cases (4.3%), 3 times in 2 cases (2.9%), and 4 times in 1 case (1.4%).

The mean maternal age was 28.55 ± 5.88, and the mean body mass index (BMI) was found to be 27.38 ± 3.96. Spontaneous pregnancy was noted in 59 cases (85.5%), while the number of intrauterine insemination and in vitro fertilization pregnancies were 2 (2.9%) and 8 (%11.6), respectively. The cigarette smoking rate was 5.8% among all cases.

Total placenta previa was observed in 40 cases, with a rate of 58%, and the remaining 29 pregnancies were complicated by low-lying placenta. Placenta was located anteriorly in 36 cases (52.2%), while 33 cases (47.8%) had posterior placenta. Cesarean delivery was performed at 37 weeks of gestation for planned cases. Uterine compression sutures, intrauterine balloon tamponade, and uterine artery ligation were performed in 7.2% ( *n* = 5), 13% ( *n* = 9), and 1.4% ( *n* = 1) of the cases, respectively.

Gestational week at birth ranged between 20 and 40 (35.9 ± 2.48) weeks, and the mean birthweight was found to be 2,719 ± 567 g. The preterm delivery rate was 43.5%, and 9 out of 30 cases were before the 34 ^th^ week of gestation. Also, PPROM was noted in 3 cases, with a rate of 4.3%. There was 1 intrauterine fetal demise (1.4%) and 11 cases of IUGR (15.9%).

There were 28 patients (40.6%) in the massive bleeding group, whereas the remaining 41 cases (59.4%) had a calculated blood loss < 1,000 mL. Red blood cell transfusion was required in 14 cases (20.3%). The majority of the cases ( *n* = 10) had been stabilized after the transfusion of 2 units. In the remaining 4 cases, more transfusion of red blood cell packages was necessary (3, 4, and 5 units of red blood cell packages in 2, 1 and 1 cases, respectively). There was no statistically significant difference in terms of maternal, fetal, and delivery characteristics between the two groups, as shown in Table 1 . Calculated blood loss and number of cases with requirement of red blood cell transfusion were statistically different between the groups (< 0.005 and 0.002, respectively).

**Table 1:** Comparison of the groups

	Non-massive bleeding ( *n* = 41)	Massive bleeding ( *n* = 28)	*p* -value
Calculated blood loss (mL)	411.52 (102–976)	1,393 (1,000–3,431)	< 0.005
Transfusion requirement	3 (7.3%)	11 (39.3%)	0.002
Age	28.02 ± 5.794	29.32 ± 6.019	0.637
BMI	27.34 ± 3.779	27.43 ± 4.290	0.438
Miscarriage history	10 (24.4%)	8 (28.5%)	0.783
Assisted reproductive technology	5 (12.2%)	5 (17.9%)	0.221
IUI	2 (4.9%)	0	
IVF	3 (7.3%)	5 (17.9%)	
Smoking	3 (7.3%)	1 (3.6%)	0.513
Placenta location			0.625
Anterior	20 (48.8%)	16 (57.1%)	
Posterior	21 (51.2%)	12 (42.9%)	
Type of previa			0.806
Placenta previa	18 (43.9%)	11 (39.3%)	
Low-lying placenta	23(56.1%)	17 (60.7%)	
Preterm delivery	17 (41.5%)	13 (46.4%)	0.806
IUGR	5 (12.2%)	6 (21.4%)	0.304
PPROM	3 (7.3%)	0	0.143
Gestational week at birth	37 (27–40)	37 (27–39)	0.904
Birthweight (grams)	2,890 (1,040–3,400)	2,765 (1,110–3,775)	0.673
Postpartum hospital stay (day)	2 (2–5)	3 (2–11)	0.369
Preoperative Hb (g/dL)	11.2 ± 1.326	11.3 ± 1.516	0.847
Preoperative Htc (%)	33.7 ± 3.851	34.4 ± 4.295	0.566

**Abbreviations** : BMI, body mass index; IUI, intrauterine insemination; IVF, in vitro fertilization; IUGR, intrauterine growth restriction; PPROM, premature rupture of membranes.

## Discussion

Placenta previa may be responsible for peripartum massive bleeding, and the risk increases with adherent placenta. Additionally, hysterectomy, blood transfusion, septicemia and thrombophlebitis are also responsible for increased maternal morbidity and mortality in these women. 10 Thus, placenta previa should be managed in an experienced center with a team consisting of an obstetrician, a pediatrician, and an anesthesiologist.

A recent systematic review reported the incidence of massive hemorrhage in women with placenta previa. They have shown that the frequency of postpartum bleeding was 22.3% in placenta previa cases. 11 On the other hand, controversial results were also given by different authors, and rates differed from the lowest (3%) to the highest (56%) in these heterogeneous studies. 12 13 Nur Azurah et al. 14 reported that postpartum hemorrhage rate was 23.4% in primigravida women without a significant difference from multigravidas. Our study found higher incidence of massive bleeding in nulliparous women with placenta previa. Among 61 women, 40.6% of the deliveries were complicated with postpartum hemorrhage. This can be explained by two scenarios. First, our hospital is a referral hospital located in the capital city of Turkey. Referred patients may be admitted to our center at a later stage, when delivery has already started. Second, the operators are not the same in all surgeries, and experience levels differ among the surgeons. Most of the elective surgeries are performed in the morning shifts after preparing blood products. A team composed of residents of perinatology and gynecology and obstetrics perform the surgery under the supervision of a maternal fetal medicine specialist. In the night shifts, there is also a team consisting of residents on duty every day.

Identifying risk factors, even in the antenatal period, to predict excessive bleeding may be useful. Several studies were conducted for this purpose. 14 15 The most important factor related with bleeding is reported to be placental adherence, with an adjusted odds ratio [OR] of 123.1 (95% Cl, 4.5–3,395.2). 15 Placenta previa in a patient with a prior caesarean section is the prominent factor for placenta accreta spectrum. Repeated cesarean sections are also known to increase the risk of placenta accreta ∼ 2.6 times. 16 Because of the worldwide increased rates of caesarean delivery, adherent placenta and related complications started to be a major health problem. However, placenta accreta spectrum is very rare in nulliparous pregnancies, and no placental attachment disorder was diagnosed in our series.

Some studies have shown that complete placenta previa is more likely related with peripartum bleeding because of the increased placental area on the cervix. 17 18 Additionally, anterior placenta is reported to be associated with increased hemorrhage compared with posterior placenta. The risk was especially important in the occurrence of a previous uterine scar. 18 19 However, a recent article has shown that the rate of postpartum massive bleeding in pregnancies with an incomplete and posterior placenta previa is 11%. 20 In the current study, we did not find a significant difference between the localization of the placenta and bleeding volume. These findings together with the previous results show that placenta previa may be complicated by excessive hemorrhage beside the type of placenta previa. Thus, all women with placenta previa should be managed carefully in the pre- and perinatal periods because of the potential excessive bleeding risk.

Maternal characteristic and ultrasonographic findings are used to create a scoring model to identify women at high risk for massive bleeding. Caesarean delivery, advanced maternal age and sponge-like appearance of the cervix were reported to be risk factors for postpartum hemorrhage in placenta previa cases. 15 Caucasian ethnicity and multiple pregnancy has also been found to be related with bleeding complications. 4 However, most of the previous studies were designed to diagnose placenta accreta spectrum disorders. On the other hand, advanced maternal age, antepartum bleeding history, anterior placenta, multiple lacunae in placenta, and increased vascularity have been shown to be related with massive bleeding in placenta previa cases, even without an adherent placenta. 21 Because of the retrospective design of our study, ultrasonographic findings could not be evaluated in the present study. It seems reasonable to use sonography carefully and use an internationally accepted standard protocol in all placenta previa cases.

Fetal and neonatal complications are another important point of placenta previa management. Previous studies revealed that the rate of preterm delivery, fetal growth restriction, and perinatal mortality has been higher in pregnancies complicated with placenta previa. 22 The histopathological evaluation of the placenta previa by Weiner et al. 23 has shown that placental vascular problems (both hemorrhage and thrombosis) are more common in placenta previa. Thus, abnormal placentation is related with impaired maternal fetal perfusion, which is most probably responsible from improper fetal growth. 23 Preterm delivery and its consequences are another issue related with placenta previa. A recent systematic meta-analysis has found that prematurity, perinatal mortality, and neonatal intensive care unit admission are increased in placenta previa cases. The preterm delivery rate is especially higher in the presence of adherent placenta and vasa previa. 24 According to our findings, preterm delivery rate is 43.5% and IUGR is 15.9% in nulliparous women with placenta previa, in accordance with the findings in the literature.

## Conclusion

Maternal excessive bleeding may complicate placenta previa pregnancies in nulliparous women. We did not find any predictive features for massive hemorrhage based on clinical features, delivery features, or placental location in primigravida patients. Thus, all women with a placenta previa diagnosis carry a risk for bleeding, regardless of their placenta location or type and maternal characteristics. Moreover, these women should be monitored in terms of preterm delivery and IUGR during antenatal care. An experienced team and blood bank are the key points to minimize the maternal and fetal adverse outcomes.
